# Lumps, Bumps, and Diagnostic Stumps: A Case Report of IgG4‐Related Disease

**DOI:** 10.1002/ccr3.73793

**Published:** 2026-10-23

**Authors:** Michael Miller, Jennifer Strouse, Nanmeng Yu, Carol Holman, Lindsay Moy

**Affiliations:** ^1^ Department of Medicine University of Iowa Health Care Iowa City Iowa USA; ^2^ Division of Immunology, Department of Medicine University of Iowa Health Care Iowa City Iowa USA; ^3^ Division of Hematology Oncology and Bone Marrow Transplantation, Department of Medicine University of Iowa Health Care Iowa City Iowa USA; ^4^ Department of Pathology University of Iowa Health Care Iowa City Iowa USA

**Keywords:** Castleman disease, generalized lymphadenopathy, histopathology, hypergammaglobulinemia, IgG4‐related disease

## Abstract

IgG4‐related disease and idiopathic multicentric Castleman disease are rare conditions that can present with overlapping features, complicating diagnosis and delaying proper treatment.

## 1 Introduction

IgG4‐related disease (IgG4‐RD) is an immune‐mediated inflammatory disorder that has the potential to have multiorgan involvement on presentation. Clinical manifestations vary broadly from localized salivary gland enlargement to renal failure secondary to postrenal obstruction from widespread retroperitoneal fibrosis [1]. A salient histopathologic feature of this disease process includes dense lymphoplasmacytic infiltrate with predominant IgG4‐positive plasma cells [2]. Other histopathologic features include storiform fibrosis and obliterative phlebitis [3]. Corticosteroid therapy is considered first‐line treatment; however, long‐term use has toxicities [1]. B cell‐depleting agents, such as inebilizumab and rituximab, and conventional synthetic disease‐modifying antirheumatic drugs, such as azathioprine or mycophenolate mofetil, may be used as steroid‐sparing maintenance therapies [1, 4].

Similar to IgG4‐RD, Castleman disease (CD) is an immune‐mediated lymphoproliferative disorder that has the potential to affect multiple organ systems. CD can be divided into unicentric and multicentric subtypes with regards to regional lymph node involvement [5]. Multicentric CD tends to present with nonspecific symptoms including fever, night sweats, and weight loss [5]. Multicentric CD can be classified by etiology—subdivided into human herpesvirus (HHV)‐8 associated; polyneuropathy, organomegaly, endocrinopathy, monoclonal gammopathy, skin changes (POEMS) syndrome associated; and idiopathic (iMCD) variants [5]. CD can also be classified by histopathologic features—two primary histologic subtypes include the plasma cell and hyaline vascular variants [6]. The plasma cell variant displays a greater degree of sheet‐like interfollicular plasmacytosis and normal to hyperplastic germinal centers. The hyaline vascular variant has more prominent vascular proliferation, and germinal centers can be atrophic [6]. Treatments target the interleukin (IL)‐6 axis; these include siltuximab and tocilizumab [5].

The following case presents an increasingly encountered diagnostic dilemma between two diseases that share similar pathologic features but require distinct treatment approaches. Both IgG4‐RD and iMCD are rare lymphoproliferative disorders that have the potential to present with multiorgan involvement and hypergammaglobulinemia. Even pathologically, these disorders may be difficult to distinguish, thus making an accurate initial diagnosis a challenge.

## 2 Case History

A 46‐year‐old man with no significant past medical history presented to his primary care provider for a 3‐month history of painless submandibular and cervical lymphadenopathy. He denied symptoms of fever, malaise, or significant weight changes. His exam lacked focal neurologic abnormalities and findings of peripheral edema. Computed tomography (CT) of the neck identified asymmetric enlargement of the left lacrimal gland, partial effacement of the right vallecula, bilateral axillary lymphadenopathy, and borderline cervical lymphadenopathy. Five months after symptom onset, the patient traveled to his home country where fine needle aspiration (FNA) of a left‐sided cervical lymph node revealed nonspecific chronic inflammatory changes and lymphocytes.

Six months after symptom onset, he had returned to the United States and was evaluated by otolaryngology. Repeat CT of the neck (Figure 1A) redemonstrated cervical lymphadenopathy and revealed slight enlargement of the lacrimal glands. There was no evidence of necrosis or infection. He exhibited no focal neurologic symptoms at interval assessments. He underwent FNA of three right‐sided cervical lymph nodes which again demonstrated nonspecific reactive lymphoid tissue. Flow cytometry and pathologic evaluation did not reveal significant abnormalities or malignancy.

**FIGURE 1:**
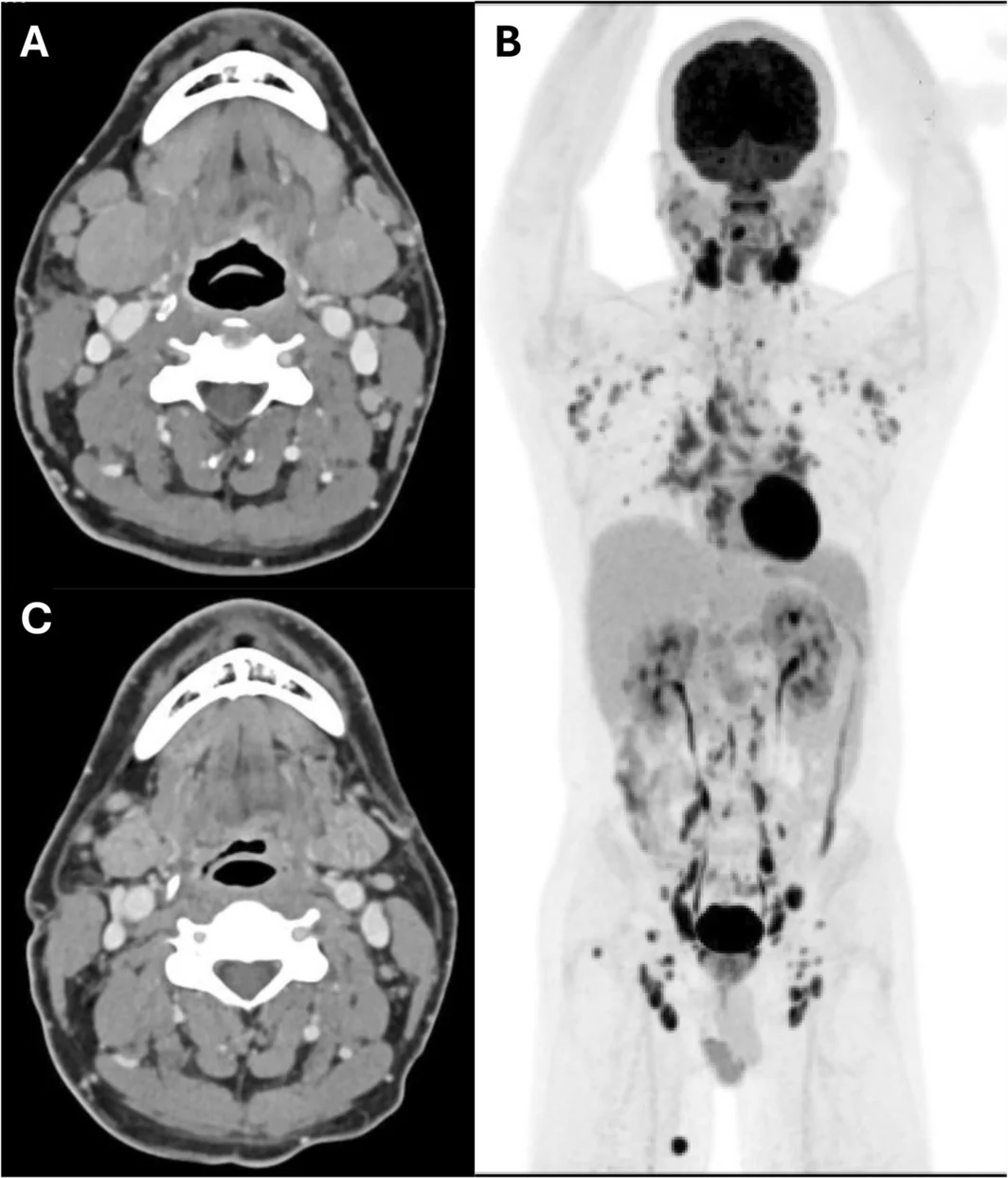
(A) CT of the neck obtained 6 months after symptom onset showing cervical lymphadenopathy. (B) PET/CT image showing widespread FDG‐avid lymphadenopathy. Notably, there is hypermetabolic lymphadenopathy noted in the cervical nodes as well as moderate uptake in enlarged parotid, sublingual, and submandibular glands. There are also avid lymph nodes in the mediastinal, axillary, paravertebral, retroperitoneal, and inguinal regions. Of note, there are subpleural and perifissural nodules in the lungs, and some subcutaneous nodules in the chest wall and thigh. (C) CT of the neck obtained after rituximab therapy showing resolution of cervical lymphadenopathy.

Given repeated nonspecific findings from FNA, he underwent excisional biopsy of the right cervical lymph node. The pathology revealed increased follicles with variably sized germinal centers that showed hyalinization and “onion skinning” of the mantle zones (Figure 2). Also observed was a dense lymphoplasmacytic infiltrate with a predominance of plasma cells, of which more than 95% stained positively for IgG4. No storiform fibrosis or obliterative phlebitis was observed. These pathologic findings were compatible with the plasma cell variant of CD. Histopathologic grading using criteria set forth by the 2017 consensus criteria showed the following: regressed germinal centers, grade 1; follicular dendritic cell prominence, grade 1; vascularity, grade 2; hyperplastic germinal centers, grade 2; plasmacytosis, grade 3 [7]. Subsequent positron emission tomography (PET)/CT identified widespread hypermetabolic lymphadenopathy above and below the diaphragm (Figure 1B).

**FIGURE 2:**
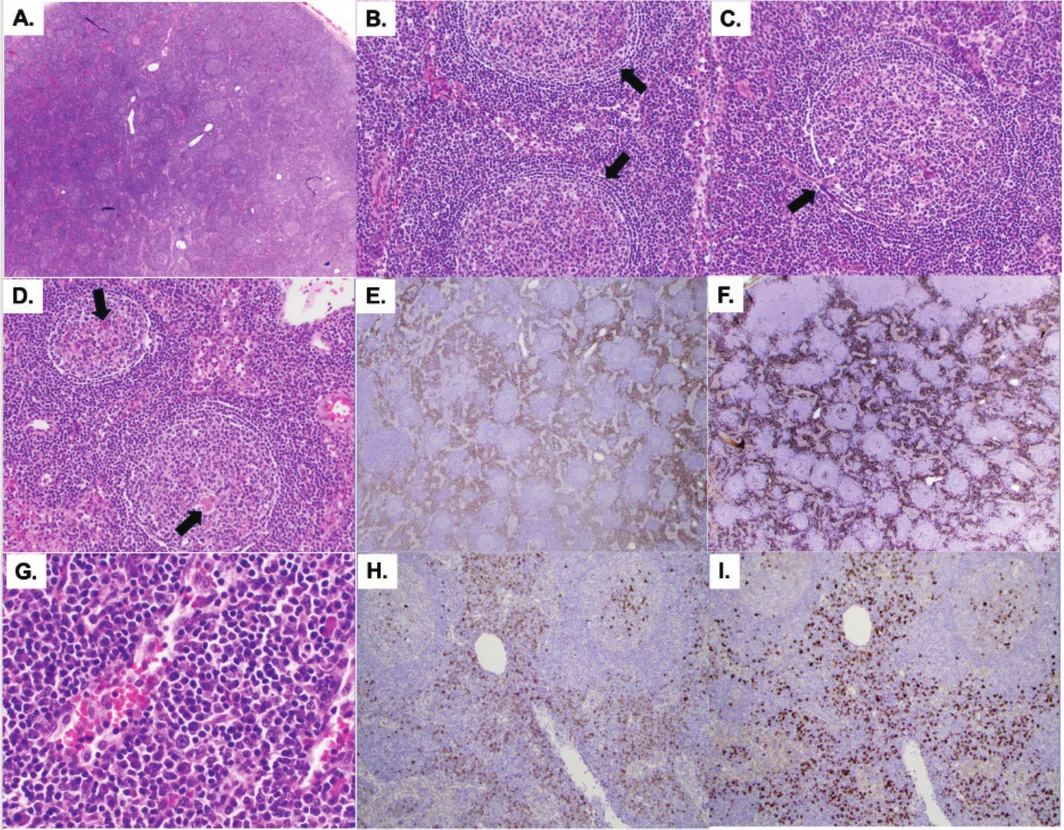
Histology and immunohistochemistry of lymph node biopsy. There are numerous follicles with variably sized germinal centers throughout the lymph node (A, H&E 2X). The mantle zones contain concentric rings of small lymphocytes, imparting an “onion skin” appearance (arrows) (B, H&E 20X), and small hyalinized vessels penetrate germinal centers (arrow) (C, H&E 20X). The germinal centers contain abundant hyalinized material (arrows) (D, H&E 20X). There are numerous interfollicular plasma cells which express CD138 (E, CD138 immunostain 2X) and IgG4 (F, IgG4 immunostain 2X). The plasma cells have mature morphologic features (G, H&E 50×) and are polytypic with immunostains for kappa (H, 10X) and lambda (I, 10X).

## 3 Investigations and Treatment

Given the concerning pathologic findings, he was referred to hematology for further evaluation. His serology was notable for multiple findings which are highlighted in Table 1. His baseline inflammatory markers were noted to have elevated erythrocyte sedimentation rate (ESR) and normal C‐reactive protein (CRP). He was additionally noted to have a mild baseline anemia, hypoalbuminemia, and significantly elevated hypergammaglobulinemia with markedly elevated IgG levels. His serum IgG4 level was not evaluated until a later follow up. As a part of his workup for MCD, his IL‐6 level was not found to be elevated, and his HHV‐8 and HIV were negative.

**TABLE 1:** Laboratory values during various timepoints of the patient's disease course.

	Initial hematology evaluation	Siltuximab #1	Siltuximab #4	Follow up	High dose corticosteroids x1 week	Rituximab #2	Rituximab #4	Off corticosteroids	Follow up	Follow up	Follow up
	Day 1	Day 14	Day 84	Day 99	Day 111	Day 118	Day 132	Day 200	Day 271	Day 325	Day 369
ESR (0–15 mm/h)	98		68	14	> 140	84	93	131	64	55	39
CRP (≤ 0.5 mg/dL)	< 0.5		1.2	< 0.5	< 0.5	< 0.5	< 0.5	< 0.5	< 0.5		< 0.5
Hgb (13.2–17.7 g/dL)	11.4	11.3	10.2	9.8	8.8	9.7	10.4	11.7			
Plt (150–400 K/MM3)	281	280	137^a^	56^a^	49^a^	241	254	313	289		
Albumin (3.4–4.8 g/dL)	3.2	3	2.7	2.7	2.5	2.7	3.2	3.6			
Cr (0.67–1.17 mg/dL)	1.19	1.18	1.43	1.54	1.14	1.16	1.02	1.25		1.24	1.24
IgG (700–1600 mg/dL)	5396	5484	6549	7212	6907	5672	2843	1980	2198	1720	1766
IgG4 (1–123 mg/dL)				5672				898		515	
IL‐6 (≤ 2.0)	< 2.0		1662.4^a^								

Abbreviations: Cr, creatinine; CRP, C‐reactive protein; ESR, erythrocyte sedimentation rate; Hgb, hemoglobin; IgG, immunoglobulin G; IL‐6, interleukin 6; Plt, platelets.

^a^ Possible effect of siltuximab.

Based on the information that was available, his presentation was thought to be most consistent with iMCD with abundant IgG4 plasma cells. Following the diagnosis, the patient received first‐line treatment for iMCD with siltuximab. He showed no clinical improvement after four cycles. Interval serologic evaluation (Table 1) identified persistently elevated ESR and normal CRP, persistent anemia, new thrombocytopenia, and worsened renal function. His measured IL‐6 level after four cycles of siltuximab therapy was also newly elevated. His new thrombocytopenia and increased level of IL‐6 were attributed to his siltuximab. Interval CT of the neck, chest, abdomen, and pelvis highlighted persistent extensive lymphadenopathy and the presence of extranodal involvement with bilateral perirenal and periureteric soft tissue, which could account for his renal function changes. The CT also highlighted new esophageal and tracheal thickening.

Given his refractory symptoms, he was referred to rheumatology for reconsideration of the diagnosis. By this time, his symptoms included sicca and increasing nasal, sinus, and ear pressures. His exam was notable for multiple firm, nontender lymph nodes in the cervical, submandibular, and inguinal regions, with nodes measuring up to four centimeters in the submandibular region. Due to concern for IgG4‐RD, he was started on high dose corticosteroids. Within 6 days of starting the prednisone, he noted improvement in his sicca symptoms and sinus congestion. He then began rituximab. His initial laboratory derangements showed improvements throughout his treatment course (Table 1).

## 4 Outcome and Follow‐Up

Following four weekly rituximab infusions, CT of the neck, chest, abdomen, and pelvis showed remarkable resolution of all his lymphadenopathy (Figure 1C). This patient was ultimately diagnosed with IgG4‐RD based on clinical features, laboratory findings, and response to treatments, despite the absence of classic histopathologic evidence. He continued to feel well and subsequently tapered off corticosteroids.

## 5 Discussion

iMCD and IgG4‐RD are both immune‐mediated disorders that can share similar features and make for a challenging initial diagnosis. Each disease process has a distinct pathogenesis and individual treatment considerations. As this case demonstrates, a reliance on histopathology alone can lead to misdiagnosis and ultimately delay patient care. Though both conditions are often characterized by their pertinent histopathologic features, the absence of these findings should not preclude the diagnosis of either disorder.

Table 2 highlights both the similarities and differences between these disorders as well as the features that our patient experienced on his initial evaluation by hematology prior to treatment with siltuximab. Regarding clinical manifestations, generalized lymphadenopathy is seen in both conditions. Constitutional symptoms, such as fevers, chills, and night sweats, are observed in approximately half of all iMCD patients, whereas fevers are not seen in IgG4‐RD [2, 8]. Lacrimal and salivary gland involvement is a common finding in IgG4‐RD and would be atypical of iMCD [9, 10]. Our patient did not experience any constitutional symptoms and had lacrimal and submandibular enlargement, which supports the diagnosis of IgG4‐RD.

**TABLE 2:** Key clinical, histopathologic, and serologic features associated with idiopathic Multicentric Castleman disease (iMCD) and IgG4‐related disease (IgG4‐RD). Pertinent findings identified in our patient at presentation are noted.

Features	iMCD	IgG4‐RD	Our patient
**Clinical**			
Generalized lymphadenopathy	Yes	Yes	Yes
Constitutional symptoms	Yes	No	No
Salivary gland involvement	Atypical	Possible	Yes
Histopathologic	“Onion skinning” appearance	Increased IgG4+ plasma cells in tissue Storiform fibrosis with or without obliterative phlebitis	Increased IgG4+ plasma cells in tissue “Onion skinning,” no storiform fibrosis
**Serologic**			
Elevated ESR	Yes	Possible	Yes
Elevated CRP	Yes	Atypical	No
Anemia	Yes	Atypical	Yes
Thrombocytopenia	Yes	Atypical	No
Hypoalbuminemia	Yes	No	Yes
Renal dysfunction	Yes	Possible	No
Hypergammaglobulinemia	Yes	Yes	Yes
Elevated IL‐6	Yes	No	No

Abbreviations: CRP, C‐reactive protein; ESR, erythrocyte sedimentation rate; IgG4‐RD, IgG4‐related disease; IL‐6, interleukin 6; iMCD, idiopathic multicentric Castleman disease.

Regarding histopathology, the spectrum of iMCD includes changes to germinal centers, prominent follicular dendritic cells, prominent vascularity, and plasmacytosis [7]. A classic triad for IgG4‐RD includes dense lymphoplasmacytic infiltrate, storiform fibrosis, and obliterative phlebitis [2]. This patient had dense lymphoplasmacytic infiltrate but lacked storiform fibrosis and obliterative phlebitis. Interestingly, it has been observed that approximately 36% of small biopsies in presumed IgG4‐RD patients lacked both findings [11].

Lastly, regarding serologic findings, elevations in inflammatory markers—ESR and CRP—can be seen in both iMCD and IgG4‐RD [12]. Furthermore, CRP can be used as a distinguishing feature between the two disorders, as persistently elevated CRP values favor MCD [13]. Anemia, thrombocytopenia, hypoalbuminemia, and elevated IL‐6 are other laboratory findings that are characteristic of iMCD [7, 14]. Our patient notably had low baseline IL‐6 and CRP levels and subsequently developed thrombocytopenia and elevated IL‐6 following targeted therapy with siltuximab. This patient's low baseline IL‐6 was not typical of the inflammatory phenotype of iMCD. The marked elevation of IL‐6 after treatment should be interpreted in the context of siltuximab therapy as measured IL‐6 may increase following IL‐6 blockade [15].

At the time of presentation, the patient's clinical and histopathologic findings fulfilled the 2017 international consensus criteria for iMCD [7], meeting both major criteria and seven of the 11 minor criteria. Based on his lymphadenopathy, plasmacytosis, and Castleman‐like histopathology, his disease process was thought to resemble the iMCD‐idiopathic plasmacytic lymphadenopathy (iMCD‐IPL) subtype [16]. Nonetheless, the clinical phenotype of iMCD significantly overlaps with other hematologic and rheumatologic conditions. Notably, the hyaline vascular and “onion‐skinning” lymph node pattern may occur as reactive phenomena to other systemic inflammatory conditions. Achieving a diagnosis of iMCD requires extensive exclusion of other diseases. Further complexity arises from therapeutic response patterns—while improvement with anti‐IL‐6 therapy lends support to the diagnosis, nonresponse does not exclude it, as only about two‐thirds of patients benefit [17]. In such scenarios, rituximab is frequently considered as a subsequent therapeutic option. The patient was ultimately diagnosed with IgG4‐RD based on clinical features, laboratory findings, and response to treatments.

To summarize and reframe our patient's initial presentation, we had a middle‐aged man present with salivary and lacrimal symptoms and lacked systemic symptoms. His pathology was significant for dense lymphoplasmacytic infiltrate that later stained positive for IgG4 plasma cells. His serology was notable for low baseline IL‐6 and CRP. Taken together, these findings strongly support IgG4‐RD; however, diagnosis is often delayed due to its ability to mimic many other disease processes.

## 6 Conclusion

When assessing and managing patients experiencing iMCD or IgG4‐RD, it is important to maintain a thoughtful and broad differential given overlapping clinical and histologic features. Integrating aspects of clinical symptoms, laboratory results, imaging, and tissue analysis can lend to an accurate diagnosis. Patients should have ongoing reassessments while on treatments as these therapeutic strategies are tailored to the underlying disease processes. Ultimately, a multidisciplinary and holistic approach can help with diagnostic accuracy and patient outcomes. This case highlights the importance of avoiding an anchoring bias even when diagnostic criteria appear satisfied.

## Author Contributions

**Michael Miller:** writing – original draft, writing – review and editing, visualization. **Jennifer Strouse:** conceptualization, data curation, supervision, writing – review and editing, writing – original draft, project administration. **Nanmeng Yu:** writing – original draft, writing – review and editing. **Carol Holman:** visualization, writing – review and editing, writing – original draft. **Lindsay Moy:** visualization, writing – review and editing, writing – original draft, conceptualization.

## Funding

The authors have nothing to report.

## Ethics Statement

IRB approval was not required. No materials from other sources were reproduced.

## Consent

Written informed consent was obtained from the patient for publication of this case report and any accompanying images. A copy of the written consent is available for review by the journal editor upon request.

## Conflicts of Interest

The authors declare no conflicts of interest.

## Data Availability

The data that support the findings of this study are available on request from the corresponding author. The data are not publicly available due to privacy or ethical restrictions.
